# Incontinence urinaire révélant une fistule vésico-utérine: à propos d’un cas

**DOI:** 10.11604/pamj.2018.31.32.15595

**Published:** 2018-09-14

**Authors:** Bounoual Mohammed, Omana Jean Paul, Ahsaini Mustapha, Tazi Karim, Mellas Soufiane, El Ammari Jalaleddine, Tazi Mohammed Fadl, El Fassi Mohammed Jamal, Farih Moulay Hassan

**Affiliations:** 1 Service d’Urologie, CHU Hassan II, Fès, Maroc

**Keywords:** Vessie, utérus, fistule vésico utérine, césarienne, Bladder, uterus, vescicouterine fistula, cesarean section

## Abstract

La fistule vésico-utérine (FVU) est une lésion rare et ne représente que 4% de toutes les fistules uro-génitales. Elle est le plus souvent consécutive à une césarienne et réalise une communication entre la vessie et l'utérus. Nous rapportons un cas d'une jeune patiente qui présentait une FVU suite à une césarienne. Le but de ce travail était d'analyser les aspects cliniques et thérapeutiques de cette affection.

## Abstract (en)

Vescicouterine fistula (VUF) is a rare lesion accounting for only 4% of all urogenital fistulas. It is most often secondary to cesarean section and forms a connection between the bladder and the uterus. We report the case of a young patient with VUF secondary to cesarean section. This study aims to investigate the clinical and therapeutic features of this disorder.

## Introduction

La fistule vésico-utérine (FVU) est une lésion rare et ne représente que 4% de toutes les fistules uro-génitales [1]. Elle est le plus souvent consécutive à une césarienne et réalise une communication entre la vessie et l'utérus [2, 3]. Sa prévalence est variable selon les pays, le diagnostic repose sur l'interrogatoire et l'examen clinique et le traitement est chirurgical [2, 3]. Le but de ce travail était d'analyser les aspects cliniques et thérapeutiques de cette affection.

## Patient et observation

**Figure 1:**
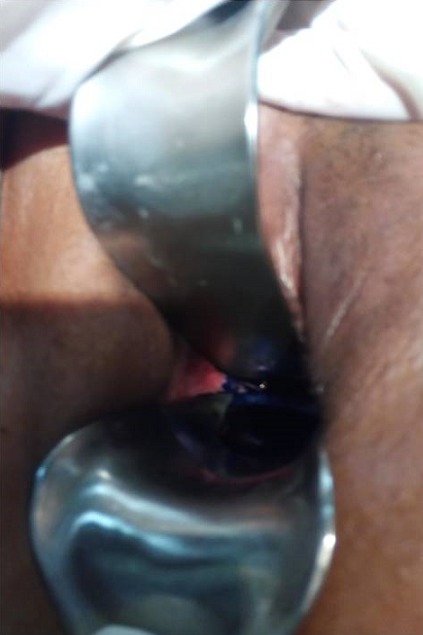
Examen gynécologique montrant issue de bleu de méthyline a travers le col utérin

**Figure 2:**
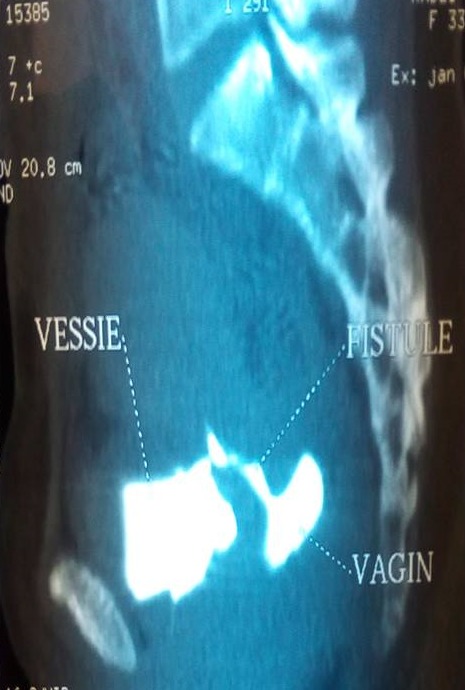
Cystoscanner montrant la fistule vésico utérine

**Figure 3:**
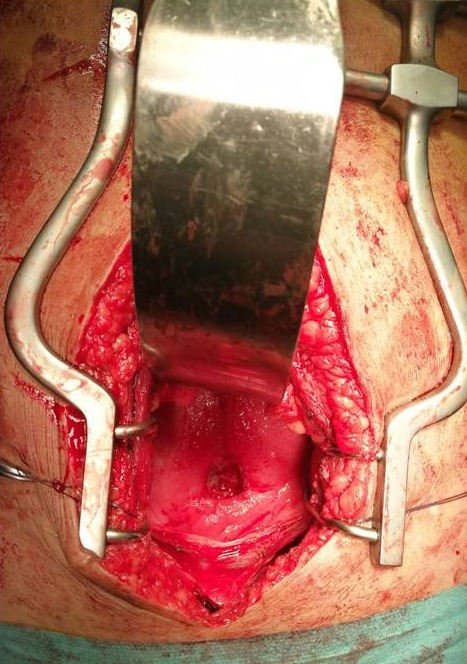
Image pér opératoire montrant une large fistule vésico utérine

Il s'agit de Madame Z. âgée de 33ans, P4G5, qui a accouché par césarienne pour rupture prématurée des membranes avec extraction d'un fœtus mort in utero en 12/2017 dans un hôpital périphérique. Les suites post opératoires deux semaines après étaient marquées par la survenue d'un hématome intra péritonéal découvert à l'échographie suite à un ballonnement abdominal, fièvre à 40^°^c et AEG. La parturiente a été reprise au 22^ème^jour post césarienne comme faisant une péritonite. Suite à l'issu des urines par le vagin, la parturiente nous sera transférée comme faisant une FVV .A notre Examen, la parturiente était lucide, cohérente avec AEG; Examen gynécologique réalisé sous valve et au toucher vaginal n'avait pas objectivé une perte des subsistances à travers la paroi vaginale, par contre issu de bleu de Méthylène par le col utérin et nous avons conclu à une fistule vésico vaginale (Figure 1). Un bilan biologique dans son ensemble était normal GB à 6600/mm^3^, la CRP à 4 mg /l, la fonction rénale correcte, sauf le résultat bactériologique notamment ECBU qui était revenu positif à un germe sensible au Céftriaxone, dont KLEBSIELLA PNEUMONIAE. Un cystoscanner (Figure 2) réalisé objectivait une large FVU. Après 10 jours de traitement au céftriaxone en raison de 2G/24 heures, ECBU de contrôle était revenu stérile et la parturiente a bénéficié d'une cure chirurgicale par la voie haute de sa FVU (Figure 3) en 2 plans utérin et vésical inversant selon la technique de CHASSAR-MOIR, technique dont le principe est le dédoublement sutures des deux plans vaginal et vésical après avivement des berges avec une sonde vésicale gardée pendant 3 semaines. Les suites post opératoires étaient simples avec bonne évolution clinique.

## Discussion

Bien que rare, la fistule vésico-utérine atteint essentiellement des femmes jeunes en période de procréation [1]. La césarienne est la principale étiologie décrite de FVU comparativement a une revue de littérature et était la seule cause présentée par Jowzik [2] qui rapporte 796 cas de FVU. Le mécanisme de son développement est lié à une plaie vésicale méconnue ou à la prise d'une portion vésicale mal décollée au cours de l'hystérorraphie [3]. D'autres étiologies sont rapportées dans la littérature comme l'accouchement dystocique [4] exceptionnelle dans les pays développés, les man'uvres instrumentales par forceps ou curetage [5], la tuberculose pelvienne [6], le myome dégénéré et plus rarement des FVU d'origine infectieuse (actynomycotiques, par exemple) après mise en place d'un stérilet inchangé depuis des années [7-9] La symptomatologie clinique est variée mais pour notre cas, elle était dominée par les fuites urinaires isolées sans hématurie cyclique comme démontré dans le syndrome de youssef qui représente une forme clinique rare portant une hématurie cyclique avec aménorrhée sans fuites urinaires et qui caractérise essentiellement les FVU haute dans laquelle l'écoulement se produit de l'utérus vers la vessie [9]. Cette forme marque l'existence d'une fistule fonctionnant dans le sens vessie-utérus décrite par Musset [10]. Devant les pertes d´urines le diagnostic de fistule vésico-vaginale, beaucoup plus fréquente, peut être évoqué. L'association FVV et FVU est possible et cela a été rapportée dans 20 à 37% des cas dans la littérature [2]. Plus rarement la FVU est découverte fortuitement au cours d'une hystérosalpingographie demandée pour des troubles gynécologiques divers [3]. L'examen gynécologique sous valve avant et après remplissage de la vessie de préférence par du bleu de méthylène permet une orientation clinique en observant la fuite du produit par le col utérin qui signe indirectement la présence de la fistule mais il existe des faux négatifs d'où le recours aux examens complémentaires. Son deuxième objectif est d'éliminer une fistule vésico-vaginale ou une incontinence urinaire vraie [3].

L'UIV constitue l'examen d'imagerie le plus pratiqué dans la littérature. Elle a double intérêt: poser le diagnostic positif de FVU en montrant au temps cystographique le passage du produit de contraste vers la cavité utérine sous la forme d'une hystérographie et surtout éliminer une lésion urétérale associée [9]. L'UCRM est réalisée généralement lorsque l'UIV est non concluante. La cystoscopie permet de visualiser l'orifice vésical de la fistule, préciser sa taille et sa localisation qui est souvent rétro-trigonal médian. L'hystérosalpingographie avec clichés de profil est exceptionnellement demandée. Le scanner et surtout le cysto-scanner constitue une imagerie alternative à l'UIV [3, 9]. L'objectif de traitement est de supprimer la communication entre la vessie et l'utérus. Dans certaines petites FVU, un drainage vésical par une sonde continu parfois aspiratif selon Ben Zineb [11, 12] permet une fermeture spontanée de ces fistules après l'involution utérine. Autre moyen thérapeutique conservateur a été décrit dans la littérature, consiste à une éléctro-coagulation par voie cystoscopique de l'orifice vésical de la fistule suivi d'un drainage par une sonde vésicale pendant deux semaines [13]. La chirurgie constitue le meilleur et le seul traitement efficace pour les fistules larges. Ce traitement est indiqué idéalement 3 mois après l'accident pour que les lésions soient bien cicatrisées et stables. La voie d'abord est une voie haute soit trans-vésicale extra péritonéale ou trans-péritonéale si on procédera à une interposition d'épiploon ou de péritoine entre le plan vésical et utérin. La technique opératoire consiste à réaliser un dédoublement vésico-utérin avec suture séparée de l'utérus et de la vessie [14, 15]. La prévention reste le meilleur moyen thérapeutique. Ceci est obtenu en respectant certaines précautions lors de césarienne: réalisation d'un bon décollement vésico-utérin, vérification de l'intégrité de la vessie et réparation des éventuelles plaies vésicales sous couverture d'un drainage vésical d'au moins 10 jours [3].

## Conclusion

La césarienne est la principale cause de la FVU. Quand cette dernière est diagnostiquée, sa prise en charge est chirurgicale. Selon notre expérience, il faut procéder par voie haute à l'excision du trajet pathologique puis à la suture vésicale inversant et à la suture utérine. Cependant le meilleur traitement reste préventif et repose sur une meilleure technique de la césarienne basée sur le trépied: mise en place d'une sonde vésicale avant tout accouchement; dissection prudente de la vessie avant toute incision utérine; recherche et réparation immédiate de toute plaie vésicale.

## Conflits d’intérêts

Les auteurs ne déclarent aucuns conflits d'intérêts.
